# Fission Yeast CSL Proteins Function as Transcription Factors

**DOI:** 10.1371/journal.pone.0059435

**Published:** 2013-03-15

**Authors:** Martina Oravcová, Mikoláš Teska, František Půta, Petr Folk, Martin Převorovský

**Affiliations:** 1 Department of Cell Biology, Faculty of Science, Charles University in Prague, Prague, Czech Republic; University of Cambridge, United Kingdom

## Abstract

**Background:**

Transcription factors of the CSL (CBF1/RBP-Jk/Suppressor of Hairless/LAG-1) family are key regulators of metazoan development and function as the effector components of the Notch receptor signalling pathway implicated in various cell fate decisions. CSL proteins recognize specifically the GTG[G/A]AA sequence motif and several mutants compromised in their ability to bind DNA have been reported. In our previous studies we have identified a number of novel putative CSL family members in fungi, organisms lacking the Notch pathway. It is not clear whether these represent genuine CSL family members.

**Methodology/Principal Findings:**

Using a combination of *in vitro* and *in vivo* approaches we characterized the DNA binding properties of Cbf11 and Cbf12, the antagonistic CSL paralogs from the fission yeast, important for the proper coordination of cell cycle events and the regulation of cell adhesion. We have shown that a mutation of a conserved arginine residue abolishes DNA binding in both CSL paralogs, similar to the situation in mouse. We have also demonstrated the ability of Cbf11 and Cbf12 to activate gene expression in an autologous fission yeast reporter system.

**Conclusions/Significance:**

Our results indicate that the fission yeast CSL proteins are indeed genuine family members capable of functioning as transcription factors, and provide support for the ancient evolutionary origin of this important protein family.

## Introduction

Transcription factors of the CSL (CBF1/RBP-Jk/Suppressor of Hairless/LAG-1) family belong among key regulators of metazoan development. They are context-dependent activators or repressors of gene expression and function as the effector components of the Notch receptor signalling pathway required for various cell differentiation-related decisions [1]–[3]. Defects in Notch/CSL signalling have been implicated in numerous human diseases including several types of cancer [4], [5]. Apart from their role in Notch signalling, Notch-independent functions in gene regulation have also been described for CSL proteins, and RBP-L, one of the two mammalian CSL paralogs, appears to operate completely independently of Notch [6]–[8].

CSL target genes typically contain a well-defined sequence motif (GTG[G/A]AA) in their regulatory regions, which is bound specifically by CSL proteins [9]–[11]. Several CSL mutants compromised in their ability to bind DNA have been reported [12] and the crystal structure of the *C. elegans* CSL bound to DNA has provided a rationale to explain the effects of these mutations and to describe the mode of DNA binding in CSL family members [13].

In our previous studies, we have identified a number of novel putative members of the CSL protein family in various fungal species [14], [15], i.e., in organisms lacking the other Notch pathway components [16]. We have shown that Cbf11 and Cbf12, the CSL paralogs in the fission yeast *Schizosaccharomyces pombe*, exert antagonistic effects on the coordination of cell cycle events and cell adhesion. Both proteins can activate gene expression in a heterologous “one-hybrid” reporter system (in the budding yeast) and Cbf11 shows a sequence-specific DNA binding activity *in vitro*, resembling closely that of the metazoan family members [17]. In spite of that, it is still not clear whether Cbf11 and Cbf12 are true CSL transcription factors [18].

In the present study we characterized further the DNA binding properties of Cbf11 and Cbf12 *in vitro* and *in vivo*, and tested their ability to activate gene expression in an autologous reporter system of *S. pombe*. Our results indicate that the fission yeast CSL proteins are indeed genuine family members capable of functioning as transcription factors, and provide support for the ancient evolutionary origin of this important protein family.

## Materials and Methods

### Yeast culture and transformation

Fission yeast cells were grown according to standard procedures [19] at 30°C in either rich medium (yeast extract with supplements; YES) or Edinburgh minimal medium (EMM; Formedium). A list of fission yeast strains used in this study is provided in Table 1. The lithium acetate method was used for transformation [20]. For thiamine-inducible overexpression of Cbf11 and Cbf12 protein variants from a plasmid, cultures were grown to mid-logarithmic phase in EMM with 15 µM thiamine (repressed state), washed twice in EMM without thiamine and grown in EMM without thiamine (induced state) for additional 22 hours before harvesting [21].

**Table 1:** Fission yeast strains used in this study.

ID	Genotype	Experiments	Source
PN559	*h^−^ ura4-D18 leu1-32 ade6-M216*	β-Gal assays, ChIP	Paul Nurse
CBF11KO	*h^−^ Δcbf11::kanR leu1-32 ura4-D18 ade6-M216*	β-Gal assays	[17]
MP03	*h^−^Δcbf12::natR ura4-D18 leu1-32 ade6-M216*	β-Gal assays	[17]
MP09	*h^−^Δcbf11::kanR Δcbf12::natR leu1-32 ura4-D18 ade6-M210*	subcellular localization, EMSA, β-Gal assays	[17]
MP15	*h^−^ cbf11-ctap4::natR ura4-D18 leu1-32 ade6-M216*	β-Gal assays, ChIP	this study
MP17	*h^−^ cbf12-ctap4::natR ura4-D18 leu1-32 ade6-M216*	β-Gal assays, ChIP	[15]

### Construction of strains and plasmids

The sequences of primers used for constructions are provided in Table 2. The fission yeast knock-in strain expressing C-terminally double TAP-tagged Cbf11 from its endogenous chromosomal locus (MP15) was constructed in an auxotrophic wild-type background (PN559) by standard PCR-mediated one-step gene tagging using the pFA6-CTAP4-natMX6 plasmid as template [22]. The mp41 forward and mp42 reverse primers contained 80 nt complementary to the genomic sequence upstream and downstream, respectively, of the *cbf11* open reading frame and 20 nt complementary to the ends of the tagging cassette. The PCR product was gel-purified, transformed into *S. pombe* cells, and nourseothricin-resistant clones in which the cassette had been integrated by homologous recombination were selected as described [22].

**Table 2:** Oligonucleotides used in this study.

ID	Sequence (5'–3')	Purpose^a^
mp41	CTTTGTTGTTCGTGATTCCAGGTGGGATTGTCATTATTGGAAAATGCGAGATTTTGCTAACGTCAAGTGCTTTTGGAAACCGGATCCCCGGGTTAATTAA	*cbf11* TAP knock-in, fwd
mp42	CCTAATTCCATCATTTTGAAAACAAATTGTATTTCAATATTTCGCCATATGAAACCACACGTAAAATTAATCATGATGCAGAATTCGAGCTCGTTTAAAC	*cbf11* TAP knock-in, rev
RBPr-fwd	CTAGACAAGGGCCGTGGGAAATTTCCTAAGCCTC	RBP reporter plasmid construction, fwd
RBPr-rev	CTAGGAGGCTTAGGAAATTTCCCACGGCCCTTGT	RBP reporter plasmid construction, rev
RBP-fwd	ACAAGGGCCGTGGGAAATTTCCTAAGCCTC	EMSA, fwd
RBP-rev	GAGGCTTAGGAAATTTCCCACGGCCCTTGT	EMSA, rev
DEL2r-fwd	CTAGACAAGGGCCCCAGCAAATTTCCTAAGCCTC	DEL2 reporter plasmid construction, EMSA, fwd
DEL2r-rev	CTAGGAGGCTTAGGAAATTTGCTGGGGCCCTTGT	DEL2 reporter plasmid construction, EMSA, rev
mp49	TATCTGCAGCTAGCGAAACATTGAAGATATATAAAGGAAGAGGAATC	reporter plasmid promoter modification, fwd
mp50	TATCCATGGAACAGGTAGTTTTC	reporter plasmid promoter modification, rev
mp71	GATGTCGACCGTCGTTTTACAACGT	*lacZ* reporter cloning, fwd
mp72	GTAGGATCCTTATTTTTGACACCAGACC	*lacZ* reporter cloning, rev
mp51	CGTCAGCCTTTATAACCatATTAATTCACAAACTGTACGTAC	*cbf11* site-directed mutagenesis, fwd
mp52	GTACGTACAGTTTGTGAATTAATatGGTTATAAAGGCTGACG	*cbf11* site-directed mutagenesis, rev
mutF	CATTGTTATGCTGAGCCCGGTAGTGATTGAATAAACAAACGC	*cbf12* site-directed mutagenesis, fwd
mutR	GCGTTTGTTTATTCAATCACTACCGGGCTCAGCATAACAATG	*cbf12* site-directed mutagenesis, rev
mt01	GCAGGATCCTAAACCTAGTCAGCTGGTAAC	construction of Cbf11(Δ1-172), fwd
mt02	GGATCCCGGGTCAGTTTCCAAAAGCACTTG	construction of Cbf11(Δ1-172), rev
mt03	GGATCCACATATGAATTGCCATTGTTTAAGC	construction of Cbf12(Δ1-465), fwd
mt04	GGATCCCGGGTTAGTGACTTTCCAAAGG	construction of Cbf12(Δ1-465), rev
map120	AAACATATGGACGGAGGATCCATGATTCC	construction of Cbf12(395-465), fwd
map105	ACCCGGGCTATGACAAAACATACTGAATCC	construction of Cbf12(395-465), rev
map92	AGAGAAAGAATGCTGAGTAGA	ChIP-qPCR from β-galactosidase reporters, fwd
map93	TACAAATCCCACTGGCTATA	ChIP-qPCR from β-galactosidase reporters, rev
mp88	AGCTGCTAGACACCTTCAAA	ChIP-qPCR, negative control, fwd
mp89	CCTACGGTCAAGAGAAAACT	ChIP-qPCR, negative control, rev

a 'fwd'–forward primer; 'rev'–reverse primer.

A list of plasmids used in this study can be found in Table 3. All plasmids for CSL overexpression were based on the pREP41/42 vector series for N-terminal EGFP, HA or MycHis tagging, which contain the medium-strength thiamine-regulated *nmt1* promoter version [23]. The Cbf11(Δ1-172), Cbf12(Δ1-465) and Cbf12(395–465) truncations were cloned by PCR using the High Fidelity PCR Enzyme Mix or Taq (Fermentas), TA or TOPO TA Cloning Kit (Invitrogen), suitable primers, and fission yeast genomic DNA or previously constructed plasmids containing full-length CSL cDNAs as templates [17]. CSL variants with a DNA binding mutation (DBM) in the beta-trefoil domain were constructed using the QuickChange II site-directed mutagenesis kit (Agilent) and the indicated primers. All new CSL variants were verified by sequencing.

**Table 3:** Plasmids used in this study.

ID	Description	Source
pMP74	β-galactosidase expression reporter with 3 RBP elements (sense-sense-antisense) upstream of a minimal promoter, constructed in pREP42EGFPN	this study
pMP88	β-galactosidase expression reporter with 3 DEL2 elements (sense-sense-sense) upstream of a minimal promoter, constructed in pREP42EGFPN	this study
pJR07	full-length *cbf11* cDNA was cloned into pREP41HAN in two steps as NdeI/SalI and SalI/BamHI fragments	this study
pMP64	after site-directed mutagenesis the SalI/BamHI fragment of *cbf11(DBM)* was used to replace the corresponding wild-type *cbf11* fragment in pJR07 (pREP41HAN vector)	this study
pMT01	*cbf11(Δ1-172)* was PCR-amplified from pJR10 and cloned into the BamHI site of pREP41HAN	this study
pJR10	full-length *cbf11* cDNA in pREP42EGFPN	[17]
pMP66	after site-directed mutagenesis the SalI/BamHI fragment of *cbf11(DBM)* was used to replace the corresponding wild-type *cbf11* fragment in pJR10 (pREP42EGFPN vector)	this study
pMT02	*cbf11(Δ1-172)* was PCR-amplified from pJR10 and cloned into the BamHI site of pREP42EGFPN	this study
pMP31	full-length *cbf12* (NdeI/BglII) was cloned into pREP41HAN (NdeI/BamHI)	this study
pMT09	after site-directed mutagenesis *cbf12(DBM)* (NdeI/BglII) was cloned into pREP41HAN (NdeI/BamHI)	this study
pMP34	full-length *cbf12* in pREP42EGFPN	[17]
pMT15	after site-directed mutagenesis *cbf12(DBM)* (NdeI/BglII) was cloned into pREP42EGFPN (NdeI/BamHI)	this study
pMP67	the NdeI/SalI fragment was excised from pMP34, the plasmid was Klenow-filled and religated to yield c*bf12(Δ1-394)* in pREP42EGFPN	this study
pMT04	*cbf12(Δ1-465)* was PCR-amplified from pMP31 and cloned into the NdeI/BamHI sites of pREP42EGFPN	this study
pMaP01	*cbf12(Δ1-394)* in pREP42MHN	[15]
pMaP05	*cbf12(395*–*465)* was PCR-amplified from genomic DNA and cloned into the NdeI/SmaI sites of pREP42EGFPN	this study

β-galactosidase reporter plasmids were derived from pREP42EGFPN. The part of the promoter upstream of the TATA box, which is responsible for the thiamine-dependent regulation of expression, was removed (up to the PstI site). The attenuated *nmt1* TATA box (“*nmt41*”) of pREP42EGFPN was then restored to the full-strength wild-type version yielding a functional minimal promoter unresponsive to thiamine (data not shown). A NheI site was then introduced 12 bp upstream of the TATA box. The *lacZ* gene was PCR-cloned from the drosophila pCasper-AUG-betaGal vector and fused in frame (SalI/BamHI) to GFP contained in the modified pREP42EGFPN vector. Finally, double stranded DNA oligonucleotides (derived from EMSA probes) with NheI-compatible overhangs were inserted into the NheI site and their number and orientation were determined by a combination of restriction digestion analysis, PCR and sequencing.

### Microscopy

Live cells overexpressing EGFP fusions of CSL protein variants were immobilized on a glass slide by a thin layer of agarose gel and subjected to fluorescence microscopy using an Olympus CellR system. Images were analysed with imageJ.

### Protein sequence analysis

Protein sequence conservation was assessed using ClustalW [24]. Nuclear localization signals (NLS) were searched for using PredictProtein [25], NLStradamus [26], and cNLS Mapper [27] with defaults settings.

### Western blotting

Proteins were separated on a 7.5% Tris-glycine polyacrylamide gel, transferred to a nitrocellulose membrane and probed with either an alkaline phosphatase-conjugated goat polyclonal anti-GFP (ab6661, Abcam; 1∶1200 dilution), mouse monoclonal anti-PSTAIRE (P7962, Sigma; 1∶8000), rabbit polyclonal anti-TAP (A00683, GenScript; 1∶1000) or mouse monoclonal anti-HA antibody (MMS-101P, Covance; 1∶1000), as appropriate. Goat-anti-mouse or goat-anti-rabbit alkaline phosphatase-conjugated secondary antibodies (170-6520, 170-6518; Bio-Rad) were used for detection, as required.

### Electrophoretic mobility shift assay (EMSA)

The analysis of DNA binding by Cbf11 and Cbf12 was described in detail previously [17]. Briefly, cells were harvested at the density of 2×10^7^ cells/ml by centrifugation, washed with STOP buffer (25 mM HEPES, 150 mM NaCl, 50 mM NaF, 1 mM NaN_3_; pH 8.0) and kept at −80°C. Native extracts were prepared in Lysis Buffer (25 mM HEPES, 0.1 mM EDTA, 150 mM KCl, 0.1% Triton X100, 25% glycerol, 1 M urea, 2 mM DTT, FY protease inhibitors (Serva); pH 7.6) by breaking the cells with glass beads in a FastPrep24 instrument (MP Biomedicals). Binding to a terminally ^32^P radiolabelled double-stranded DNA probe containing a canonical metazoan CSL binding site ('RBP' probe; [17]) or a mutated site ('DEL2' probe) was performed in Shift Buffer (25 mM HEPES, 34 mM KCl, 5 mM MgCl_2_; pH 7.6) and detected as a slow-migrating band on a large native 5% polyacrylamide gel. The sequences of oligonucleotide probes used in this study are given in Table 2.

For supershift experiments, DTT was omitted from the Lysis Buffer. Cell extracts (230 µg per reaction) were incubated with 1 µg of either a mouse monoclonal anti-GFP (A-11120, Invitrogen) or rabbit polyclonal anti-TAP antibody (A00683, GenScript) for 1 hour on ice, followed by 20 min incubation with the 'RBP' probe on ice.

### β-galactosidase assay

Cells harbouring the respective CSL overexpression and/or reporter plasmids were harvested from liquid cultures, washed in Z-buffer (60 mM Na_2_HPO_4_.7H_2_O, 40 mM NaH_2_PO_4_.H_2_O, 10 mM KCl, 1 mM MgSO_4_, 50 mM β-mercaptoethanol; pH 7.0), resuspended in Z-buffer and broken with glass beads in a FastPrep24 instrument. Freshly dissolved ortho-nitrophenyl-β-D-galactopyranoside (ONPG) was added to the cell lysates to a final concentration of 0.47 mg/ml and reactions were incubated at 30°C with shaking. Reactions were stopped by the addition of 0.37 volume of 1 M Na_2_CO_3_, centrifuged at 13,000×g at room temperature for 3 minutes to remove cell debris, and β-galactosidase activity was determined by measuring the absorbance at 420 nm.

### Chromatin immunoprecipitation

Fifty millilitre cultures of CSL-TAP knock-in strains were grown in EMM to the density of 1.2×10^7^ cells/ml, fixed with 1% formaldehyde for 30 min, quenched with glycine, washed with water and broken with glass beads in Lysis Buffer (50 mM HEPES, 1 mM EDTA, 150 mM NaCl, 1% Triton X-100, 0.1% sodium deoxycholate, FY protease inhibitors (Serva); pH 7.6) using the FastPrep24 machine. Chromatin extracts were sheared with the Bioruptor sonicator (Diagenode) to yield DNA fragments of ∼200 bp. The resulting chromatin extracts were used for immunoprecipitation directly with magnetic IgG beads (cat. no. 110.41, Invitrogen). The precipitated material was washed twice with Lysis Buffer, Lysis 500 Buffer (50 mM HEPES, 1 mM EDTA, 500 mM NaCl, 1% Triton X-100, 0.1% sodium deoxycholate; pH 7.6), LiCl/NP-40 Buffer (10 mM Tris-HCl, 1 mM EDTA, 250 mM LiCl, 1% Nonidet P-40, 1% sodium deoxycholate; pH 8.0), once in TE (10 mM Tris-HCl, 1 mM EDTA; pH 8.0), and eluted in Elution Buffer (50 mM Tris-HCl, 10 mM EDTA, 1% SDS; pH 8.0). Cross-links were reversed overnight at 65°C, samples were treated with DNase-free RNase followed by proteinase K, and purified using phenol-chloroform extraction and sodium acetate precipitation. The enrichment of specific target DNA sequences (the reporter plasmid promoter or a negative control genomic region) in the immunoprecipitated material was determined by quantitative PCR (qPCR) using the MESA GREEN qPCR MasterMix Plus for SYBR (Eurogentec) and the LightCycler 480 II instrument (Roche). The negative control region corresponds to nucleotides 1928274-1928359 on chromosome I, i.e., around the transcription start site of the SPAP14E8.05c gene, a region where no CSL binding could be detected *in vivo* (our unpublished data). The sequences of primers used for qPCR are given in Table 2.

## Results

### The low-complexity N-termini of Cbf11 and Cbf12 are important for their nuclear localization

**Figure 1:**
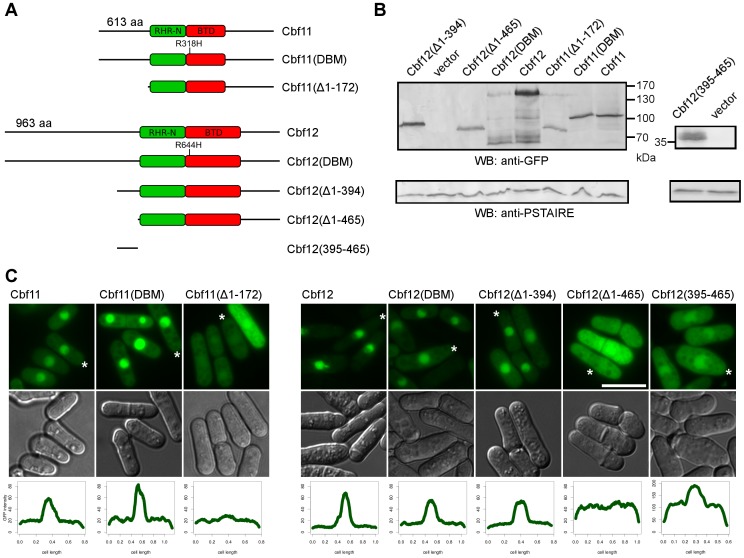
The N-termini of Cbf11 and Cbf12 are important for their nuclear localization. (**A**) Schematic representation of all Cbf11 and Cbf12 variants used in this study. The N-terminal Rel homology region ('RHR-N', green), beta trefoil domain ('BTD', red), and position of the point mutation affecting DNA binding activity are indicated. (**B**) A western blot confirming that all N-terminally EGFP-tagged CSL variants are expressed and produce a band of the expected size. The anti-PSTAIRE (Cdc2) western blot serves as a loading control. (**C**) All EGFP-CSL derivatives localize to the nucleus with the exception of variants devoid of the whole unstructured N-terminal region. Scale bar is 10 µm. The bottom panel shows longitudinal fluorescence intensity profiles of the cells marked with an asterisk.

We have previously shown that the long, likely intrinsically disordered N-termini of fission yeast CSL proteins are enriched in potential regulatory motifs, such as phosphorylation sites, and that amino acids 1–394 of Cbf12 affect negatively the ability of the protein to bind DNA *in vitro* [15]. To gain more insight into the functional significance of the protein sequences preceding the core CSL domains, we have constructed truncated versions of Cbf11 and Cbf12 lacking the whole N-terminal parts upstream of the RHR-N domain (Cbf11(Δ1-172) and Cbf12(Δ1-465); Fig. 1A). The truncated CSL variants were cloned into a vector for inducible GFP-tagged expression and their production in cells was checked by western blotting (Fig. 1B).

Fission yeast CSL proteins are hypothesized to function as transcription factors and, accordingly, full-length Cbf11 and Cbf12 localize to the nucleus [17]. Therefore, we checked whether their subcellular localization was affected by removing their complete pre-core N-termini. As shown in Fig. 1C, in contrast to their full-length counterparts, Cbf11(Δ1-172) and Cbf12(Δ1-465) were distributed almost evenly throughout the cell. Interestingly, the nuclear localization of the previously reported truncation mutant Cbf12(Δ1-394) was retained and Cbf12(395-465) directed partial nuclear accumulation when fused to GFP. Since the removal of pre-core N-termini resulted in pan-cellular localization of Cbf11 and Cbf12 (i.e., including the nucleus), other region(s) of the proteins may also contribute to their nuclear localization. We attempted to find bioinformatically potential nuclear localization signals in Cbf11 and Cbf12 (see Materials and Methods) but did not find any, with the sole exception of a monopartite NLS predicted in the N-terminus of Cbf11 (aa 121-130) by cNLS Mapper. Thus, the fission yeast CSL proteins require their unstructured N-termini for proper nuclear localization by as yet unidentified mechanism. In Cbf12, the major region affecting localization seems to be between amino acids 395–465. This region is not conserved between Cbf12 and Cbf11 or other fungal CSL proteins [15].

### Full-length Cbf12 exerts weak but specific DNA binding activity *in vitro*

The residues and peptide motifs important in metazoan CSL family members for sequence-specific DNA binding are conserved in both Cbf11 and Cbf12 [13], [14]. However, while we could detect specific and direct binding to the canonical CSL response element on DNA ('RBP' probe) for Cbf11, we were unable to demonstrate such binding activity for full-length Cb12 but only for the Cbf12(Δ1-394) truncation mutant [15], [17]. We have previously postulated a hypothesis that proteolytic removal of the inhibitory N-terminus of Cbf12 might be required *in vivo* for the protein to bind to DNA. Another possible explanation, not mutually exclusive, could be that the *in vitro* conditions of the EMSA assay are suboptimal for Cbf12 DNA binding and that increasing Cbf12 concentration might result in detectable DNA binding activity.

**Figure 2:**
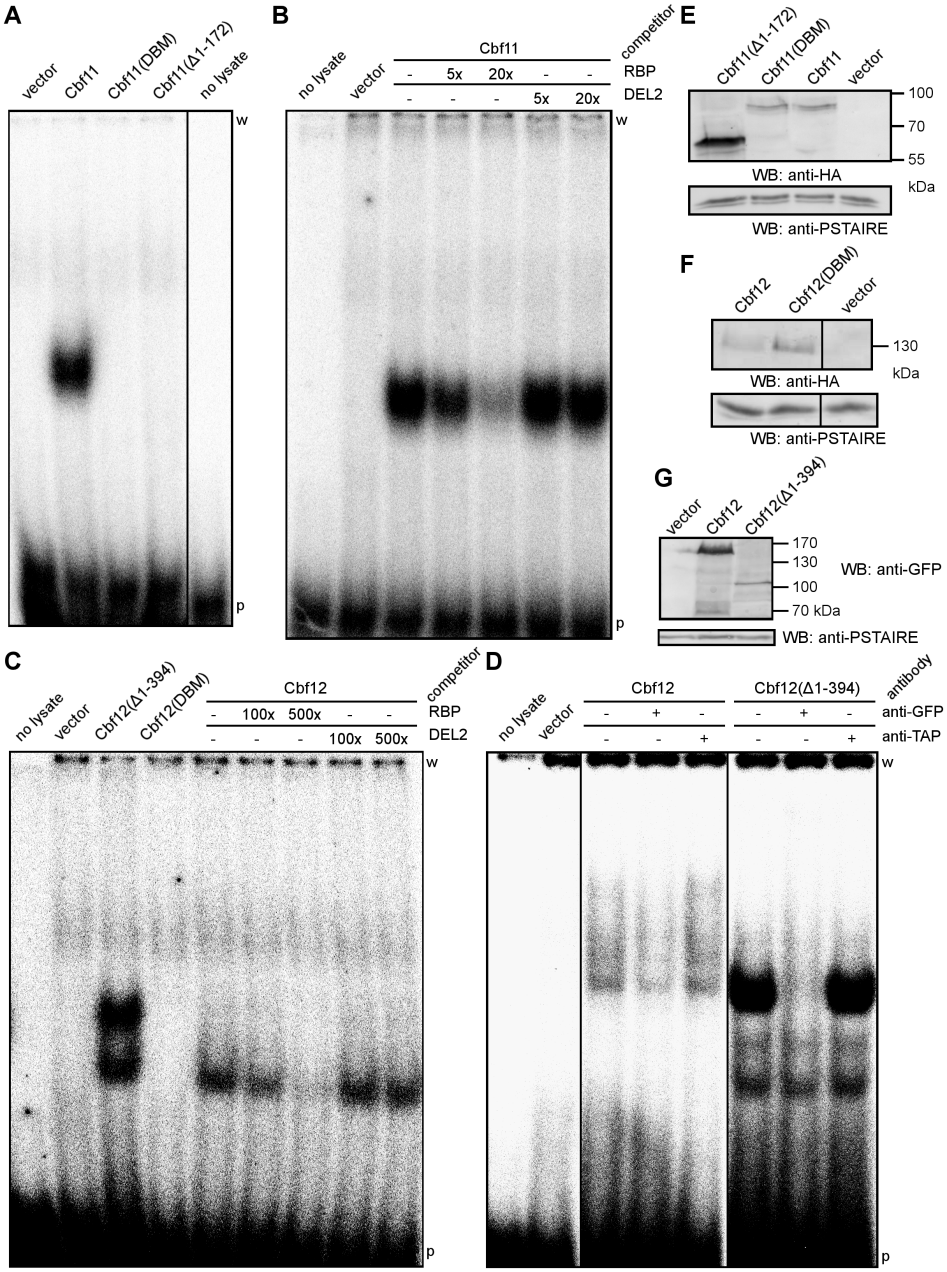
Mutation of a conserved arginine residue abolishes CSL DNA binding activity *in vitro*. (**A**) Substitution of the arginine residue at position 318 with histidine ('DBM') or a complete removal of the unstructured N-terminus abrogate the binding of Cbf11 to a DNA probe ('RBP') with the canonical CSL response element in an EMSA experiment. (**B**) The EMSA band corresponding to the DNA binding activity of the full-length non-mutated Cbf11 can be competed with an excess of the unlabelled RBP probe but not with the mutated DEL2 probe. (**C**) Full-length non-mutated Cbf12 displays very weak but specific DNA binding activity towards the RBP probe in EMSA. The Cbf12 band shift can be competed with an excess of the unlabelled RBP probe but not with the mutated DEL2 probe. No DNA binding activity was observed when the arginine residue at position 644 was substituted with histidine ('DBM'). A lane with low amounts of Cbf12(Δ1-394), previously shown to bind the RBP probe [15], is shown for comparison (n.b., during native electrophoresis, protein migration speed is not simply proportional to protein mass since native charge and shape also play important roles). (**D**) Supershift experiments with GFP-tagged Cbf12 variants. For both full-length Cbf12 and Cbf12(Δ1-394) the addition of an anti-GFP antibody, but not an anti-TAP antibody, interferes with protein-DNA complex formation. 'w'–wells; 'p'–free probe. (**E, F, G**) Western blots of cell extracts from (A), (C) and (D), respectively, confirming that all N-terminally HA-tagged and GFP-tagged CSL variants were expressed properly and that equal amounts of cell extracts were used.

To test such possibility, we have conducted EMSA experiments with increased amounts (230 µg/reaction vs. the regular 84.6 µg/reaction; 2.7-fold increase) of cell lysates from the *Δcbf11 Δcbf12* strain overexpressing full-length Cbf12 from a plasmid. Indeed, we could see a band shift of the RBP probe that was absent from the empty vector control, and that could be specifically competed with an excess of the unlabelled RBP probe but not with the DEL2 probe containing a mutated CSL response element (Fig. 2C). This Cbf12-dependent DNA binding activity was very weak compared to that of Cbf11 (cf. Fig. 2B; note different competitor concentrations).

To further ascertain that the observed band shift was produced by full-length Cbf12, we have conducted supershift experiments with N-terminally GFP-tagged Cbf12 and Cbf12(Δ1-394) (Fig. 2D, G). Three bands of different intensities appeared in the lanes with GFP-Cbf12(Δ1-394), possibly reflecting the presence of different posttranslational modifications or proteolytic products. While we did not observe any supershifted bands, the formation of the major protein-DNA complex was completely blocked by the addition of an anti-GFP antibody, but not an unrelated anti-TAP antibody, suggesting that this complex requires the GFP-Cbf12(Δ1-394) protein with an uncleaved N-terminus. The lanes with extracts from cells overexpressing full-length GFP-Cbf12 also contained multiple, rather smeary bands. Their intensity was reproducibly and specifically reduced upon the addition of an anti-GFP antibody, suggesting that these protein-DNA complexes contain or depend upon GFP-Cbf12 with an uncleaved N-terminus. Based on these results and the behaviour of the Cbf12(DBM) mutant (see below) we conclude that both fission yeast CSL proteins are able to specifically bind to the canonical metazoan CSL response element on DNA *in vitro*.

### Mutation of a conserved arginine residue abolishes DNA binding activity in Cbf11 and Cbf12

It has been reported that the substitution of the conserved arginine at position 218 to histidine dramatically reduced the DNA binding activity of the murine RBP-Jκ CSL protein without negatively affecting protein stability [12]. This residue within the BTD domain, which is also conserved in fission yeast CSL proteins, was later shown to be directly involved in contacting DNA [13]. To gain further support for the hypothesis that Cbf11 and Cbf12 are genuine CSL family members, we decided to test the effects of this point mutation (DNA binding mutant, 'DBM') in fission yeast CSLs.

To this end we have introduced the analogous R318H and R644H substitutions to Cbf11 and Cbf12, respectively, using site-directed mutagenesis (Fig. 1A). The resulting Cbf11(DBM) and Cbf12(DBM) proteins were found to be expressed and both localized to the cell nucleus (Fig. 1B, C).

When tested for binding to a DNA probe containing the canonical CSL response element (RBP probe) by the EMSA assay, wild-type Cbf11 displayed strong binding activity, as reported previously [17]. By contrast, Cbf11(DBM) showed no detectable DNA binding activity (Fig. 2A). This was not due to a lack of Cbf11(DBM) expression or its degradation (Fig. 2E). Intriguingly, no DNA binding activity could be detected for Cbf11(Δ1-172) either. Similar results were obtained with Cbf12(DBM) (Fig. 2C). In spite of the fact that the mutant protein was expressed at higher levels than wild-type Cbf12 (Fig. 2F), no band shift was detected. Thus the DNA binding activities of both the murine and fission yeast CSL proteins depend on the same conserved arginine residue in the beta trefoil domain, suggesting that the mechanism of binding is conserved in these phylogenetically distant organisms.

### Cbf11 and Cbf12 activate reporter gene expression in *S. pombe*

**Figure 3:**
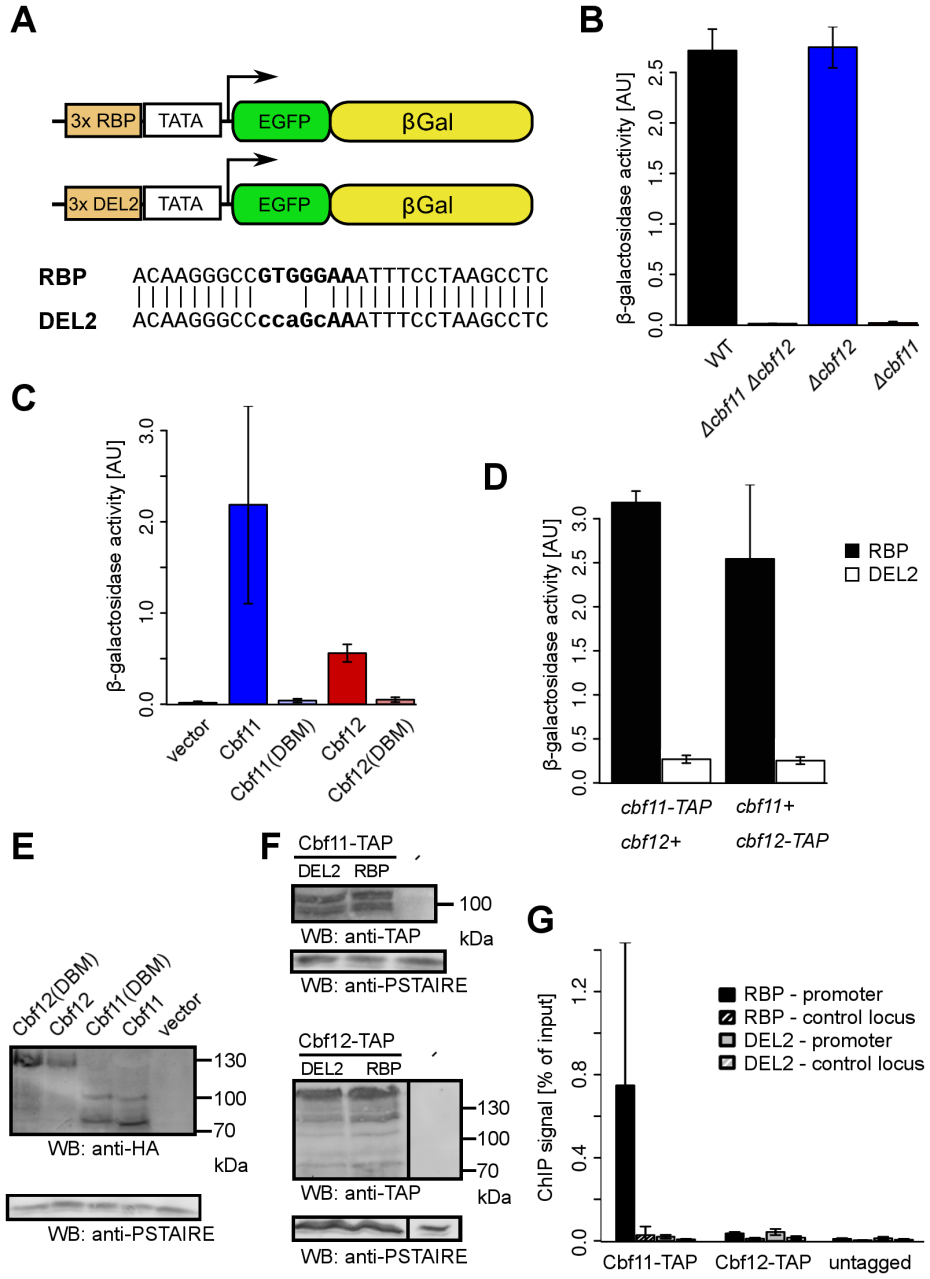
Cbf11 binds to and activates a reporter gene *in vivo*. (**A**) Schematic representation of expression reporter plasmids with the β-galactosidase gene under the control of a minimal promoter with three copies of either the canonical ('RBP') or mutated ('DEL2') CSL response element (not to scale). (**B**) β-galactosidase activity in wild-type and CSL deletion mutant strains harbouring the RBP reporter plasmid. Activation of the reporter is dependent on endogenous Cbf11 but not Cbf12. (**C**) β-galactosidase activity in the *Δcbf11 Δcbf12* strain harbouring the RBP reporter plasmid and overexpressing the indicated CSL protein variants (HA-tagged) from a plasmid. The presence of high levels of both Cbf11 and Cbf12 activates, to a different degree, the RBP reporter. This activation is abolished in both CSL proteins by the 'DBM' point mutation in the BTD domain. (**D**) β-galactosidase activity in strains with chromosomally TAP-tagged Cbf11 or Cbf12 harbouring either the RBP or mutated DEL2 reporter plasmid. Both strains show RBP reporter activation similar to the untagged wild-type strain in (B) and no activation of the DEL2 reporter with mutated CSL binding sites. (**E, F**) Western blots of cell extracts from strains used in (C) and (D), respectively, confirming that all N-terminally HA-tagged and C-terminally TAP-tagged CSL variants were expressed properly. (**G**) Chromatin immunoprecipitation of TAP-tagged Cbf11 and Cbf12 from strains described in (D). Cbf11 binds strongly the promoter region of the RBP reporter but not the mutated DEL2 reporter. No binding to either reporter could be detected for Cbf12. All data represent mean values±standard deviations from at least three independent experiments.

Both fission yeast CSL paralogs can activate gene expression when fused to unrelated DNA binding domains in a heterologous budding yeast reporter system [17]. Whether Cbf11 and Cbf12 could also act as regulators of gene expression in the autologous settings of *S. pombe* is not known. To find the answer to this question we first constructed reporter plasmids (Fig. 3A) in which the *lacZ* gene, encoding β-galactosidase, was put under the control of a minimal promoter preceded by three copies of either the RBP probe, known to be bound by fission yeast CSL proteins *in vitro*, or three copies of the mutated DEL2 probe, which is not bound (see Fig. 2B, C).

When the RBP reporter construct was introduced into wild-type fission yeast cells, strong β-galactosidase activity was detected in cell lysates (Fig. 3B). Subsequent analysis of CSL single and double deletion mutant strains showed that this reporter gene activation was fully dependent upon Cbf11 (Fig. 3B), suggesting that normal physiological levels of Cbf11 but not Cbf12 can indeed support gene activation in fission yeast.

When wild-type or DBM mutant variants of Cbf11 and Cbf12 were overexpressed in a CSL double deletion strain harbouring the RBP reporter both CSL proteins were able to trigger β-galactosidase production, although the level of reporter activation was much stronger for Cbf11 (Fig. 3C, E). Notably, no reporter activation was observer for either Cbf11(DBM) or Cbf12(DBM), indicating that DNA binding activity is required for the CSL proteins to exert their effects on gene regulation.

### Cbf11 binds to the canonical CSL response element *in vivo*

To test whether Cbf11 and Cbf12 are present physically at the promoter region of the reporter plasmids, we employed chromatin immunoprecipitation (ChIP) from cells expressing C-terminally TAP-tagged CSL alleles from their natural chromosomal loci. We first confirmed that both tagged strains supported levels of RBP reporter activation similar to untagged wild-type cells (Fig. 3D, F; this activation is fully Cbf11-dependent in the untagged strain, cf. Fig. 3B). As expected, no activation was observed for the DEL2 reporter containing mutated CSL binding sites in its promoter.

The ChIP results were in agreement with the data on reporter gene activation (Fig. 3G). Cbf11 was found to bind strongly to the promoter region of the RBP reporter, but not to the promoter region of the mutated DEL2 reporter or to a control genomic locus. No DNA binding was detected for Cbf12. Thus, under physiological protein levels during exponential growth in minimal medium, Cbf11 behaves as a sequence-specific DNA binding activator of transcription.

## Discussion

In this study, we characterized the mechanism of DNA binding of Cbf11 and Cbf12 *in vitro* and *in vivo*, and demonstrated their ability to activate gene expression in *S. pombe*. Our results strongly suggest that the fission yeast CSL proteins are genuine family members capable of functioning as transcription factors. Our data support previous notions of ancient evolutionary origin of the CSL family in the last common ancestor of metazoa and fungi [14], [16].

In our previous work, we have repeatedly failed to detect binding of full-length Cbf12 to the canonical CSL response element [15], [17]. This finding was puzzling since (i) the motifs and amino acid residues required for DNA binding in metazoan CSL family members are conserved in Cbf12, (ii) its paralog, Cbf11, shows high affinity for the CSL response element. Notably also, Cbf12 undergoes proteolytic cleavage (or degradation) *in vivo*, yielding N-terminally truncated protein isoforms, and the Cbf12(Δ1-394) truncation mutant shows weak but specific DNA binding to the CSL response element [14], [15], [17].

By using large amounts of lysates from cells overexpressing Cbf12 in the present study, we were able to demonstrate *in vitro* a sequence-specific DNA binding activity also for the full-length Cbf12 protein (or its potential physiological proteolytic product containing an uncleaved N-terminus). However, the Cbf12 binding to the RBP probe, which contains the canonical CSL response element, was very weak compared to Cbf11. It is possible that the *in vitro* conditions of our EMSA assay are suboptimal for Cbf12 to bind to the DNA probe. We consider such explanation less likely as we also failed to detect any *in vivo* DNA binding for Cbf12 expressed at physiological levels by either ChIP or a reporter gene activation assay. Notably, Cbf12 could support limited reporter activation when overexpressed, suggesting that it does possess the ability to function as an activator of transcription. Unfortunately, we were unable to get robust ChIP signals for N-terminally tagged CSL proteins overexpressed from a plasmid to confirm the Cbf12 DNA binding activity *in vivo* (data not shown). The RBP probe, which is bound strongly by Cbf11, may thus simply represent a low-affinity target for Cbf12 and therefore is bound weakly *in vitro* and does not support robust promoter activation *in vivo* under physiological Cbf12 levels. It should be noted that variant binding sites have been reported for metazoan CSL proteins, e.g. [28], [29]. Alternatively, despite possessing weak (or residual) DNA binding activity, Cbf12 may be recruited to DNA indirectly as part of a protein complex containing other DNA-binding subunits [30]. Cbf12 could also exert its effects by directly or indirectly antagonizing Cbf11 at target loci, or acting as a repressor under some conditions. Such activities could have been missed in our reporter system. These possibilities are not mutually exclusive and each of them may apply for particular endogenous target loci *in vivo*. Studies are under way to clarify this issue.

The lack of detectable binding to DNA in the DBM mutant CSL variants reported in this study, which mimics the R218H DBM mutation of the murine CSL protein, clearly demonstrates that the mode of DNA binding is likely conserved between metazoan and fission yeast family members. However, it is not known whether binding to DNA is required for CSL proteins to exert their impact on cell cycle regulation and cell adhesion in fission yeast. Thus, the Cbf11(DBM) and Cbf12(DBM) separation-of-function mutants, unable to bind DNA but otherwise unaffected in localization or stability, shall prove instrumental to answering this important question.

There are more open questions to be addressed by future studies: What are the endogenous target genes of the fission yeast CSL transcription factors and what are the dynamics of their regulation by Cbf11 and Cbf12? Are endogenous CSL target genes activated, similar to the *lacZ* reporter gene, or repressed *in vivo*? What protein partners mediate CSL functions? And what are the signals and upstream regulators acting upon fission yeast CSL proteins? Interestingly, both Cbf11 and Cbf12 are phosphorylated at multiple sites, mostly in their N-terminal regions, by yet unidentified kinase(s) ([15] and data not shown). Furthermore, protease(s) targeting the unstructured N-termini of fission yeast CSL paralogs may hypothetically play important roles in the regulation of CSL subcellular localization and DNA binding activity (this study and [15]). While this manuscript was in preparation, a study by Wells and colleagues [31] showed that Zfs1, an RNA binding protein of the tristetraprolin family of CCCH tandem zinc finger proteins, binds directly to the 3' UTR of *cbf12* mRNA and causes its destabilization. Loss of *zfs1* resulted in increased cell-cell adhesion, largely due to the upregulation of *cbf12*. The authors also proposed some direct Cbf12 target genes related to cellular adhesion [31]. We are currently mapping systematically the genome-wide CSL DNA binding profiles and interrogating the transcriptomes of *Δcbf11* and *Δcbf12* mutant cells to obtain a comprehensive picture of the biology of the fission yeast CSL transcription factors.
